# Disparities in the Prevalence of Osteoporosis and Osteopenia in Men and Women Living in Sub-Saharan Africa, the UK, and the USA

**DOI:** 10.1007/s11914-023-00801-x

**Published:** 2023-06-23

**Authors:** Kate A. Ward, Camille M. Pearse, Tafadzwa Madanhire, Alisha N. Wade, June Fabian, Lisa K. Micklesfield, Celia L. Gregson

**Affiliations:** 1 MRC Lifecourse Epidemiology Centre, University of Southampton, Southampton General Hospital, Southampton, SO16 6YD UK; 2 MRC Unit The Gambia, London School of Hygiene and Tropical Medicine, Banjul, The Gambia; 3 Department of Infectious Disease Epidemiology, Faculty of Epidemiology and Population Health, London School of Hygiene & Tropical Medicine, London, UK; 4 The Health Research Unit Zimbabwe, Biomedical Research & Training Institute, Harare, Zimbabwe; 5 MRC/Wits Rural Public Health and Health Transitions Research Unit, School of Public Health, Faculty of Health Sciences, University of the Witwatersrand, Johannesburg, South Africa; 6 SAMRC/Wits Developmental Pathways for Health Research Unit, School of Clinical Medicine, Faculty of Health Sciences, University of the Witwatersrand, Johannesburg, South Africa; 7 Musculoskeletal Research Unit, Bristol Medical School, University of Bristol, Bristol, UK

**Keywords:** Ageing, Africa, DXA, Osteoporosis, Bone density, Treatment

## Abstract

**Purpose:**

To review the rising prevalence of osteopenia and osteoporosis in sub-Saharan Africa and the challenges this poses to governments and healthcare services. Using existing studies, we compare the prevalence of osteopenia and osteoporosis in men and women from sub-Saharan Africa to US and UK cohorts. Context-specific disparities in healthcare are discussed particularly the challenges in diagnosis and treatment of osteoporosis.

**Recent Findings:**

There are few epidemiological data describing the burden of osteoporosis in sub-Saharan Africa. In the studies and cohorts presented here, osteoporosis prevalence varies by sex, country and area of residence, but is generally higher in African populations, than has previously been appreciated. Risk factors contributing to poorer bone health include HIV, malnutrition and “inflammaging.”

**Summary:**

Reprioritization towards care of ageing populations is urgently required. Equitable access to implementable preventative strategies, diagnostic services, treatments and pathways of care for bone health (for example embedded within HIV services) need now to be recognized and addressed by policy makers.

**Supplementary Information:**

The online version contains supplementary material available at 10.1007/s11914-023-00801-x.

## Scope of the Problem

In low- and middle-income countries (LMIC), there are already more than 1 billion people aged over 60 years [1]. Older people in LMICs spend longer living with disability and dependence than do those in high-income settings, impacting individuals, families, communities and healthcare systems in some of the most resource-poor countries [2]. Life expectancy is rising more rapidly in Africa than any other continent globally, with a 200–300% rise in the population aged 60 years and older predicted over coming decades compared to less than 100% rise in Europe and Northern America [1]. The United Nations declaration of the Decade of Healthy Ageing 2021–2030 states that there has never been a timelier opportunity to act to ensure the health of ageing adults, their families and communities [3].

In sub-Saharan African (SSA) countries, the shifting demographics, together with rapid urbanisation and changing dietary and physical activity patterns, are generating an exponential rise in the prevalence of non-communicable diseases (NCDs) [4]. Osteoporosis and fragility fractures are a major, and growing, contributor to the rise in NCDs, with a relative doubling of high fracture risk in men and women in Africa between 2010 and 2040 [5], with musculoskeletal diseases overall accounting for more years lost due to disability than cancer and cardiac disease combined [6]. Furthermore, in parts of SSA, chronic infections (e.g. HIV), with both short- and longer-term sequelae, continue to affect millions of people every year. The successful roll-out of antiretroviral therapy (ART) now means HIV can be considered a chronic disease of ageing. The long-term effects of a life living with HIV in SSA are still largely unknown; however, data suggest negative impacts on musculoskeletal health through immuno-senescence, low-grade systemic inflammation and premature ageing [7•, 8, 9]. Furthermore, exposure to specific antiretroviral drugs further increases fracture risk [10].

Healthcare systems in SSA have prioritised provision of maternal and child healthcare, and infectious diseases of which HIV and TB have been primary foci. Now, these systems need to expand to include management of chronic care of long-term conditions, often within the context of multimorbidity. The hitherto unrecognised healthcare needs of a growing ‘older generation’, perhaps inevitably risk exacerbating health inequalities for ageing populations. For example, early studies from 1960s South Africa led to the false belief that fragility fractures were not seen in Black African populations [11]. More recent, robust evidence has dispelled this myth [12]; osteoporotic fractures of the hip and spine are increasingly reported in countries such as South Africa [13••, 14, 15], where a doubling of fracture rates is predicted over coming decades [16••]. In African women in South Africa, for example, hip fracture incidence was 176/100,000 in White, 147.7/100,000 in Indian and 43.5/100,000 in Black, women, rates similar to other transitioning populations across the world [13••, 17]. Access to diagnosis including medical specialities and dual energy X-ray absorptiometry (DXA) scanning is limited. Similarly common treatments for osteoporosis are not widely available, potentially explained by lack of inclusion on the WHO Essential medicine list and hence omission from national pharmacy lists, meaning provision is limited to a small proportion of the population, without sufficient financial means. Hence, there is an urgent need in SSA to raise awareness of age-associated osteoporosis and future fragility fracture risk, to individuals, patient advocacy groups, healthcare providers, government stakeholders and policy makers.

The purpose of this review is therefore to focus on studies from three diverse African countries, South Africa, The Gambia and Zimbabwe. Data are compared to US population-based and UK cohort data, to understand differences by country, sex, age, race and rural versus urban living. Finally, this review discusses barriers to osteoporosis diagnosis and treatment in SSA and the potential implications in terms of future fracture risk for populations as they continue to age.

## Studies and Methodology

Data collected between 2010 and 2019 were collated and reviewed from seven published studies to enable comparison of adult populations, aged 40 years and older, across five countries: The Gambia, South Africa, Zimbabwe, the UK and the USA. All African populations were Black African; the US Health ABC (Health, Aging and Body Composition) study included Black/African American and White American populations (ethnicity was not determined in Health ABC), whilst the UK Hertfordshire Cohort Study (HCS) included only White European (non-Hispanic) (Table 1). Whilst not generalizable to the whole continent, the data here provide representation of cohorts in countries of diverse geography, HIV prevalence and stage in economic and epidemiological transition. In terms of economic status, defined by Development Assistance Committee (DAC) listing [18], African cohorts spanned least developed (The Gambia), lower-middle income (Zimbabwe) and upper middle–income countries (South Africa), with a mix of rural- and urban-dwelling African populations in West and Southern Africa. In 2020, health expenditure as a proportion of GDP was, by country, South Africa 8.58%, The Gambia 2.61% and Zimbabwe 3.43% [19]; by comparison, the figure was 18.8% and 11.4% in the USA and UK respectively at this time. Access to private healthcare happens in the wealthier minority, with the public health system providing all care to 71% and 70% of the population in Zimbabwe and South Africa, and 96% of the population in The Gambia [20–22]. For South African studies, participants with HIV were retained in the analysis to ensure the populations were representative of the whole study population. Because the Zimbabwe study was a case–control design, we analysed data from control participants who did not have HIV. For Health ABC and Hertfordshire, we retained all participants irrespective of bisphosphonate use; a sensitivity analysis in the Health ABC study showed exclusion of those reporting ever use of bisphosphonates made little difference to the results. Notably, no bisphosphonate use was reported by any participant in any of the African studies. The following studies/cohorts were used, and data from baseline and follow-ups included maximising the sample (Tables 1 and 2):

1. The Gambian Bone and Muscle Ageing Study (GamBAS), a prospective study of men and women, recruited in sex-stratified 5-year age bands (40 + years), from a rural demographic surveillance site in The Gambia, in West Africa [23, 24•, 25]
2. The Zimbabwean Menopause study, a cross-sectional study conducted in Harare, women aged 40–60 years were enrolled from local communities around the central hospital in the city [26••].
3. The Agincourt Health and Socio-Demographic Surveillance System (HDDS), in South Africa, a population-based sample of participants sampled from a rural community located in the North-East of South Africa (men and women age 21–80 years were recruited; however, only those aged 40 years and older were included in the current analyses) [27].
4. The Middle-Aged Soweto Cohort (MASC), in South Africa, a longitudinal study conducted at the Chris Hani Baragwanath Hospital in Soweto, Johannesburg. Baseline data collection (2011–2015) recruited 1004 randomly selected caregivers of the Birth to Twenty Plus cohort. A randomly selected sub-sample of women aged 40–61 years were followed-up (January 2017 to August 2018) [26••, 28]
5. The Hertfordshire Cohort Study (HCS), in the UK, a prospective, population-based study of the lifecourse origins of adult disease among community-dwelling men and women, recruited adults born in Hertfordshire, UK 1932–1939 [29]
6. The Health, Aging and Body Composition Study (HealthABC) in the USA, with more than 7-year prospective cohort study, recruited Black and White Americans (age 68–89 years) at baseline (in 1997–1998) [https://healthabc.nia.nih.gov/] [30]

**Table 1:** Descriptive summary of women across all studies

	Country	The Gambia		South Africa		South Africa		Zimbabwe		UK		USA		USA	
	Study	GamBAS		Agincourt^a^		MASC		Menopause study ^b^		HCS		HealthABC Black		HealthABC White	
	Location	Rural		Rural		Urban		Urban		Urban		Urban		Urban	
Study design		Prospective cohort		Prospective cohort		Prospective cohort		Cross-sectional		Prospective		Prospective		Prospective	
*n* participants^*c*^* (HIV*^*d*^*)*		249		547 *(129)*		450 *(65)*		393 *(193)*		468		615		782	
Race^d^		Black African		Black African		Black African		Black African		White British		Black		White	
Ethnicity^e^		79.9% Mandinka, Fula 16.2%, Jola 2.4%, Other 1.3%		South Eastern Bantu — predominantly Tsonga		36% Zulu, 18.3% Sesotho, 12.5% Tswana, 3% Other		100% Shona							
*n* measurements		455		418		760		200		1049		2463		3308	
Mean (SD) age		61.4 (11.9)		45.0 (14.8)		52.5 (6.3)		49.6 (5.8)		69.5(5.9)		77.0 (4.1)		77.7 (4.1)	
Median [IQR] BMI		21.3 [19.2–23.9]		30.0 [25.1–34.9]		33.0 [29.0–37.3]		30.3[26.8–34.5]		26.7 [23.8–30.3]		29.0 [25.2–33.2]		25.6 [ 22.7–28.7]	
		*n*	Mean (SD)	*n*	Mean (SD)	*n*	Mean (SD)	*n*	Mean (SD)	*n*	Mean (SD)	*n*	Mean (SD)	*n*	Mean (SD)
Total hip T-score	40–49 y	90	0.18 (1.22)	82	0.42 (1.09)	276	0.41 (1.18)	100	0.53 (1.16)	–	–	–	–	–	–
	50–59 y	125	− 0.99 (1.30)	87	− 0.10 (1.30)	385	0.12 (1.32)	100	0.34 (1.41)	–	–	–	–	–	–
	60–69 y	120	− 1.87 (0.81)	51	− 0.32 (1.17)	99	− 0.44 (1.13)	–	–	632	− 0.65 (1.11)	29	− 0.48 (1.27)	28	− 1.52 (1.10)
	≥ 70 y	120	− 2.37 (0.98)	28	− 0.98 1.32)	–	–	–	–	417	− 0.89 (1.22)	2428	− 1.08 (1.38)	3276	− 1.84 (1.12)
Femoral neck T-score	40–49 y	90	0.71 (1.04)	82	0.43 (1.19)	276	− 0.60 (1.14)	100	− 0.18 (1.15)	–	–	–	–	–	–
	50–59 y	125	− 0.35 (1.28)	87	− 0.25 (1.30)	385	− 1.07 (1.23)	100	− 0.67 (1.25)	–	–	–	–	–	–
	60–69 y	120	− 1.14 (0.81)	51	− 0.63 (0.99)	99	− 1.67 (1.03)	–	–	632	− 1.15 (1.02)	29	− 0.83 (1.32)	28	− 1.68 (0.93)
	≥ 70 y	119	− 1.67 (0.88)	28	− 1.41 (1.32)	–	–	–	–	417	− 0.86 (1.19)	2432	− 1.27 (1.82)	3279	− 2.11 (0.96)

^a^Agincourt participants were restricted to those aged 40 and over

^b^Only those without HIV included in this comparison study

^c^For the prospective cohorts, the baseline number of participants is stated

^d^HIV only stated where recorded, in MASC these were self-report and not verified. In Agincourt and Menopause

^e^Ethnicity was determined by language spoken and self-report. The figures stated for GamBAS are from the West Kiang Demographic Surveillance System data. For MASC, from the Awigen cohort. The Agincourt population are South–Eastern Bantu predominantly Tsonga-speaking REFS

**Table 2:** Descriptive summary of men across all studies

	Country	The Gambia		South Africa		UK		US		US	
	Study	GamBAS		Agincourt ^a^		HCS		HealthABC Black		HealthABC White	
	Location	Rural		Rural		Urban		Urban		Urban	
*n* participant^b^* (HIV*^*c*^*)*		239		258 *(34)*		498		446		846	
Mean (SD) Age		61.1 (12.3)		43.8 (16.3)		69.0 (6.6)		77.2 (4.0)		77.9 (4.1)	
Median [IQR] BMI		20.4 [18.8–22.5]		24.2 [21.4–27.9]		26.8 [24.6–29.1]		26.8 [23.9–29.9]		26.6 [24.5–29.2]	
*n* measurements		404		224^a^		1067		1743		3517	
		*n*	Mean (SD)	*n*	Mean (SD)	*n*	Mean (SD)	*n*	Mean (SD)	*n*	Mean (SD)
Total hip T-score	40–49 y	91	0.60 (1.14)	34	0.95 (1.17)	–	–	–	–	–	–
	50–59 y	115	0.33 (0.93)	30	0.88 (1.40)	13	0.99 (1.06)]	–	–	–	–
	60–69 y	94	− 0.39 (1.18)	41	0.64 (1.40)	651	0.59 (1.16)]	22	0.92 (1.26)]	32	0.04 (1.04)]
	≥ 70 y	104	− 0.52 (1.33)	12	0.47 (1.38)	403	0.48 (1.32)]	1716	0.29 (1.37)]	3477	− 0.32 (1.30)]
Femoral neck T-score	40–49 y	91	0.94 (1.19)	34	0.63 (1.16)	–	–	–	–	–	–
	50–59 y	115	0.49 (0.98)	30	0.40 (1.26)	13	− 0.20 (0.873)]	–	–	–	–
	60–69 y	94	− 0.28 (0.98)	41	− 0.01 (1.48)	651	− 0.34 (1.06)]	22	0.16 (1.11)]	32	− 0.81 (1.07)]
	≥ 70 y	104	− 0.54 (1.32)	12	− 0.23 (1.38)	403	0.19 (1.31)]	1720	− 0.46 (1.25)]	3482	− 1.15 (1.16)]

^a^Agincourt participants were restricted to those aged 40 and over

^b^For the prospective cohorts, the baseline number of participants is stated

^c^HIV data only collected in Agincourt

All studies included measures of femoral neck and total hip BMD by DXA, enabling T-score calculation following the International Society for Clinical Densitometry (ISCD) 2019 Official Positions, and National Osteoporosis Foundation of South Africa (NOFSA) guidelines, which advises, for derivation of T-Scores in Black African populations, the use of US National Health and Nutrition Examination Survey (NHANES) III White female reference data from 20 to 29 years-olds. [31] (supplementary information)). For consistency, we calculated T-scores for all adults aged 40 years and older, rather than Z-scores for those aged under 50 years old, as is recommended by the ISCD. Total hip DXA data were the most complete across the studies, whilst femoral neck is the recommended clinical site for BMD measurement; hence, data from both sites were included. Tables 1 (women) and 2 (men) show race, ethnicity (where available) and number of participants and measurements included mean age, median body mass index (BMI) and mean total hip and femoral neck T-scores for each study.

**Fig. 1:**
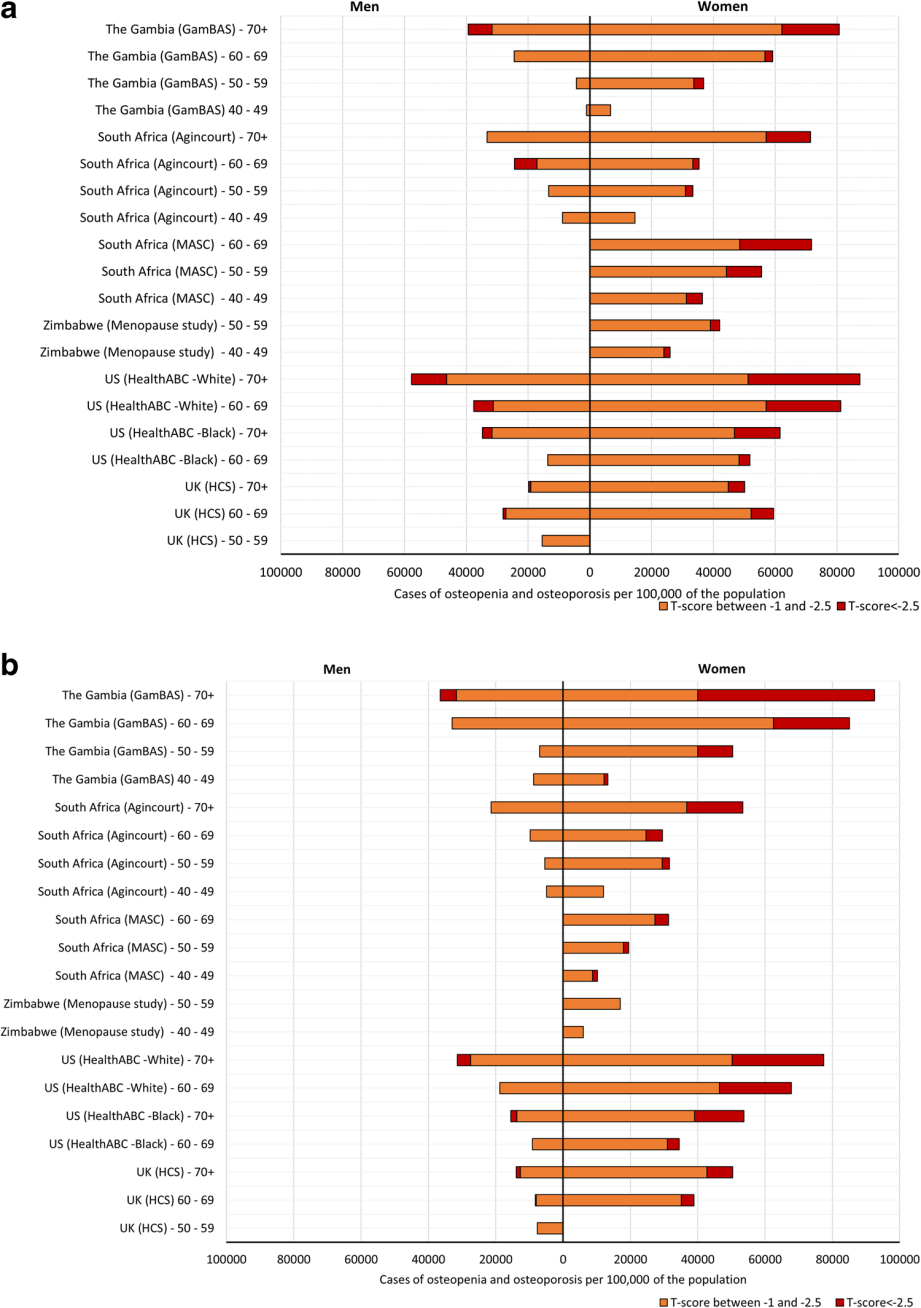
Population prevalence of **a** femoral neck osteoporosis and osteopenia and **b** total hip osteoporosis and osteopenia, per 100,000 population

**Fig. 2:**
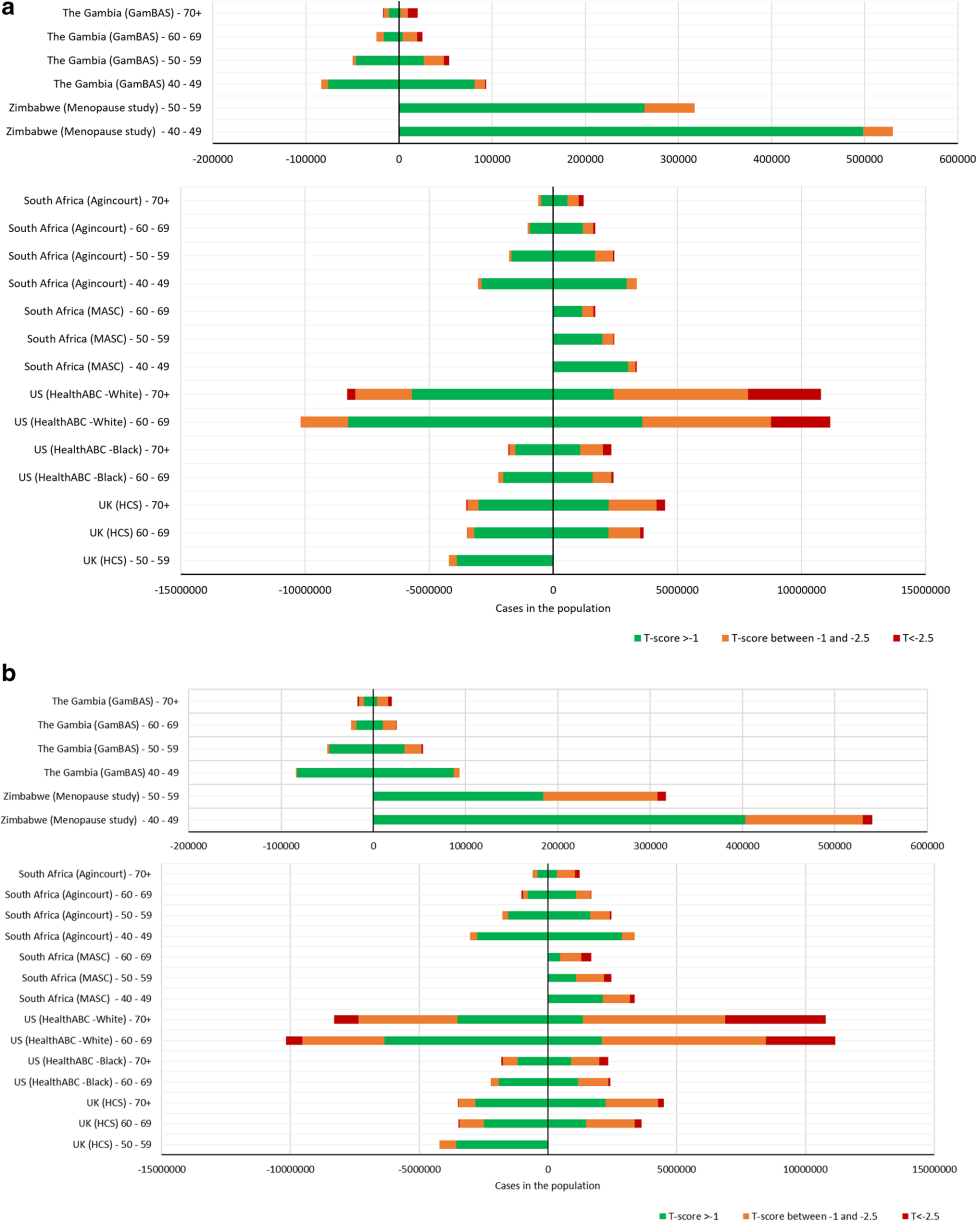
Country-specific burdens of **a** femoral neck osteoporosis and osteopenia and **b** total hip osteoporosis and osteopenia. NB, men on left of the 0 line, women on right

Figure 1 a (femoral neck) and b (total hip) show the prevalence of osteopenia, defined as a T-score between − 1 and − 2.5, and osteoporosis, defined as a T-score less than or equal to − 2.5, per 100,000 people, calculated by sex and 10-year age bands for each study population. Then, Fig. 2 a (femoral neck) and b (total hip) apply these prevalence to the contemporaneous United Nations 2015 population estimates, to estimate the total number of men and women living in each study country with normal BMD, osteopenia and osteoporosis [32].

## Prevalence of Osteoporosis and Osteopenia

### *Femoral Neck (*Fig. 1a*, Supplementary Table 1a)*

The prevalence of osteoporosis at the femoral neck was similar across the rural populations in Gambian and South African women and similar in prevalence to US Black women. In urban South Africa, osteoporosis prevalence was the highest of all the African studies, which was comparable to the prevalence in US White women and much higher than in US Black and UK White women. Urban dwelling Zimbabwe women had a two to fourfold lower prevalence of osteoporosis than urban South African women.

The population prevalence of osteopenia at the femoral neck followed a similar pattern to that of osteoporosis in women in rural populations of The Gambia and South Africa. Across all Black African populations, osteopenia prevalence was more similar to US and UK White women, than US Black women.

In men, prevalence of osteopenia and osteoporosis was approximately one-third lower than those in women in their corresponding countries. In general, across all populations examined, osteoporosis was uncommon in men under the age of 70 years. In 60–69-year-old men in rural South Africa and The Gambia, the prevalence was similar to that of US White men in the same age group.

### *Total Hip (*Fig. 1b*, Supplementary Table 1b)*

In Gambian women, the population prevalence of osteoporosis was much higher than in women in all other countries. The lowest osteoporosis prevalence was reported in White UK women. In contrast, for osteopenia, the population prevalence was similar in The Gambia, South Africa, UK and US Black and White populations. Prevalence of osteopenia was lower in urban-dwelling women in South Africa and Zimbabwe.

Total hip osteopenia and osteoporosis were less common in men than women across all studies. In men, osteoporosis at the total hip was rare. The population prevalence for osteopenia in men was again highest in Gambian Black African and White American-men, being lowest in UK and Black American men.

### *Country-Specific Differences in Prevalence of Osteoporosis and Osteopenia (*Fig. 2a, b*; Supplementary table 2a, 2b)*

Osteoporosis and osteopenia are common in SSA women and to a lesser extent SSA men; with the exception of The Gambia, femoral neck osteoporosis is more prevalent than total hip across the cohorts (Fig. 2a, b). Of the five countries reviewed, the US White population is greatest in size and the number of people living with impaired bone health, whilst there are more urban-dwelling Black South African women living with femoral neck osteoporosis (between 60 and 69 years *n* = 392,193), than among Black US women (between 60 and 69 years *n* = 84,859). In fact, over the age of 60 years, fewer than half of women in urban South Africa have a normal femoral BMD T-Score, a population with an estimated HIV prevalence of 19.1% [33]. In Zimbabwe, women over 30% of women aged 50–59 years have osteopenia at the femoral neck, and importantly, this does not include women living with HIV. Most women in The Gambia over the age of 60 years have a total hip T-score less than − 1.0 (i.e. in the osteopenic or osteoporotic range). The UK prevalence of either femoral neck or total hip osteoporosis is surprisingly low, which is likely due to a healthy survivor effect in the cohort, or that we did not exclude those taking bisphosphonates.

## Potential Explanations for Between-Country Differences in Osteoporosis and Osteopenia Prevalence

There are several factors that may explain the country and sex differences we are seeing. Here, we have chosen three countries with varying contexts; generalizability of course cannot be assumed, but there are key observations and commonalities in risk factors that should be considered within SSA. These include a rising prevalence of multimorbidity and increased longevity, increasing prevalence of non-communicable diseases, malnutrition (under- and over-weight micronutrient deficiency) and rapid transition to urbanisation that may change physical activity and lifestyle habits such as increased access to public transport, changes in work patterns from subsistence farming to office-based jobs, access to fast-food, smoking and in some cases alcohol consumption.

1. HIV. In SSA, chronic HIV infection continues to affect millions of people. In 2021, of the 38.4 million people around the world living with HIV, 20.6 million were living in East and Southern Africa [34]. HIV programs have been highly successful at rolling-out anti-retroviral treatment towards 95–95-95^1^ targets [35]; however, such programs do not currently routinely assess or manage the longer-term comorbidities that arise during a life lived with HIV, and a growing number of people are now living with HIV into older age [12]. We have recently demonstrated that women living with HIV in South Africa, who transition through menopause, have augmented post-menopausal bone loss [26••], compared with women who do not have HIV, highlighting the need to consider bone health as part of long-term HIV care. Osteoporosis is a silent disease, until suddenly a fragility fracture occurs; therefore, implicit in its treatment is the need to invest in preventative services.
2. Nutrition. The 2018 and 2020 Global Nutrition Report’s highlighted the unacceptably slow progress in tackling all forms of malnutrition, be it prevalence of under/overweight either alone or in conjunction with micronutrient deficiency. This also included the limited progress towards diet-related NCDs across the lifecourse and lack of quality data in older adults [36, 37]. Changing lifestyles and food availability are contributing to altered dietary patterns, to reductions in dietary diversity, and to poor micronutrient status, including inadequate vitamin D and calcium intakes. There is a growing, but often conflicting, evidence-base supporting importance of micronutrients and dietary patterns for musculoskeletal health of older adults, and importantly, limited data exist in ageing adults from across race and ethnic groups across the globe, not least in SSA [38–40]. Variability in the assessment of diet quality, food security and patterns mean findings to date are inconsistent. Ensuring it is possible to give context-specific dietary advice and understanding the impact of changes in weight with ageing and micronutrient needs of ageing populations in diverse populations is an unmet need.
3. Inflammaging. Ageing, high adiposity, chronic HIV, recurrent infections and malnutrition induce ‘inflammaging’, a state of chronic inflammation associated with ageing [41]. All these components have been associated with poor musculoskeletal health, through common inflammatory or metabolic risk factors which suggest shared aetiological mechanisms [41–43]. However, the potentially shared aetiological mechanisms that underlie inflammation-associated bone loss in different ethnic groups and contexts require specific study.

### Reference Data to Derive T-Scores

The ISCD have developed proxy-guidance for the diagnosis of osteoporosis in countries where reference data are not available [44]. To enable the cross-country comparisons, we have used a single-approach for calculation and use of T-scores for both men and women aged 40 years and above; T-scores were calculated using NHANES III data from White women aged 20–29 years. It is important to note that for clinical individual diagnostic purposes, there are specific guidelines for those aged 50 years and under, but given that very little bone loss occurs prior to the age of 50, comparing pre-menopausal women and men to the young adult reference will make little intrinsic difference to results. Taking our pragmatic approach, there are clearly sex and ethnic differences in the prevalence of osteoporosis and osteopenia between SSA and the UK and US populations. However, to fully appraise these disparities, country- and context-specific reference data are required to fully understand disease prevalence. Whilst the single approach we have taken, as per ISCD recommendation, does allow between-country comparison, there are other factors that should be considered in this approach, such as racial and ethnic differences in body composition and body size which cannot be accounted for using the currently available data. An example of this is in Zimbabwean women who had lower weight and BMI per decade age group than the NHANES reference population, leading authors to conclude that adjustments for body composition in calculation of Z-scores would be an appropriate approach [45]. In the studies included in this review, there are clear between country differences in BMI for women, where Gambian women have the lowest and Southern African women the highest BMIs. There were fewer differences among men, but again, the mean BMI was much lower in Gambian men than in other populations. Collection of country-specific reference data, at least in some SSA countries, would permit the assessment of the suitability of current guidelines for the diagnosis of osteoporosis, potentially enabling a move away from the necessity to use NHANES III reference data.

### Barriers to Diagnosis of Osteoporosis in Sub-Saharan Africa

Barriers to osteoporosis diagnosis include insufficient clinical awareness, which we hope to in part address in this review, as well as unmet clinical training needs due to a lack of specialist physicians (for example, South Africa has one geriatrician per 275,000 older people [46]; Zimbabwe and The Gambia at the time of writing have none). A further barrier stems from insufficient access to DXA scanning facilities. The data reviewed here were collected in research sites with good access to DXA scanning, but this is far from standard and does not represent what is happening across the continent. There are few data detailing availability of DXA scanners; data from 2011 showed in Morocco provision was 0.6 DXA scanners per million, while it was 5 per million in Tunisia [47]. The costs of DXA tests can also be prohibitive, costing 1/3 to ½ of average annual income in some cases [47]. Widespread DXA scanning provision is not practical in resource-constrained public healthcare settings; hence, methods of non-specialist fracture risk assessment should be a priority.

As most people who sustain a fragility fracture have a femoral neck BMD T-Score greater than − 2.5 [48], i.e. not in the osteoporotic range and classified as either osteopenic or normal, consideration of the many clinical risk factors besides BMD, for fragility fracture risk, is key. Until recently, lack of access to fracture risk assessment tools was a barrier; however, progress is certainly being made following the publication of age-, sex- and ethnicity-specific hip fracture incidence rates for South Africa [13••], which have been used to calibrate both a South African fracture risk assessment tool called FRAX™ [49•], and by proxy a Zimbabwean FRAX tool. The FRAX™ tool takes a set of pre-determined risk factors and calculates the 10-year probability of an individual’s risk of hip or major osteoporotic (clinical spine, forearm, hip or shoulder) fracture. Furthermore, hip fracture incidence data from Botswana (Southern Africa) [50], Ethiopia (East Africa) [51] and Tunisia (North Africa) [52]^2^ have enabled FRAX calibration in these settings. Notably, no data are yet available for a country in West Africa, which is where The Gambia is located. FRAX allows fracture risk assessment with or without the need for a BMD measurement, which certainly increases accessibility to bone health assessment, even where DXA provision is poor. However, there is a gap in the evidence base regarding our understanding of FRAX clinical risk factors, beyond age, T-score and prior fracture [49•], in SSA populations, as well as the role of additional context-specific risk factors that may be particularly relevant, such as HIV infection [12]. Future research is needed to validate these new FRAX tools in SSA populations, and future policy work is needed to deploy these effectively within regional guidelines and practice [53].

A further challenge in rural and peri-urban regions is lack of access to diagnostic and subsequent treatment options, given limited-service provision, such as pharmacies, physiotherapy and laboratory and radiology services. Patients will often need to travel long distances to access care [54]. Often, older people will move back to their original places of residence in rural communities once they retire meaning access to care in rural communities is a key gap in care provision currently.

### Barriers to Treatment of Osteoporosis in Sub-Saharan Africa

Barriers to treatment include insufficient specialist services, competing priorities within stretched healthcare systems, lack of access to anti-osteoporosis medicines and lack of awareness amongst healthcare providers, and policy makers. There are insufficient rheumatologists and endocrinologists, particularly with an interest in bone health, in Africa; for example, in Nigeria, there are 30 rheumatologists serving 200 million people and in Ghana 2 for 28 million people [54]. Currently, there are gross global disparities in access to anti-osteoporosis medicine. The most common osteoporosis treatments are the oral bisphosphonates. Generic oral weekly alendronate costs approximately 12 US dollars per annum, yet it is not routinely available in most public hospitals in SSA. This largely reflects the lack of prioritisation of osteoporosis medicines by the WHO Essential Medicines list, which includes not one osteoporosis medication, not even menopausal hormone treatment for women, which is very effective at reducing fracture risk [55]. Paradoxically, the WHO Essential Medicines list includes the intravenous bisphosphonate zoledronate, making this available to treat cancer-related skeletal events, but the very same drug is not listed to reduce fragility fracture risk, where the evidence base is strong [56].

A further inequality in SSA stems from differences in provision within public and private health care services where access to medicines, used commonly in high-income countries for primary and secondary fracture prevention, is only possible in the private healthcare system. Where private healthcare plans exist in South Africa, osteoporosis is not considered a primary medical benefit; hence, there is no incentive to assess and treat fracture risk. In South Africa, those with more comprehensive medical insurance are reimbursed in the case of severe osteopenia, osteoporosis and fracture [47]. Equitable access to affordable osteoporosis treatment should be a priority for health care providers and policy makers.

A further barrier to accessing osteoporosis care arises from medical pluralism — common across Africa, but particularly exemplified by traditional bone setters in West Africa. Anti-osteoporosis medicines are not promoted by traditional healers and complexities in pathways to care may prevent delay in treatment of fragility fractures.

### Fragility Fractures in Sub-Saharan Africa

The ultimate clinical manifestation of osteoporosis is a fragility fracture. One of the limitations of some of the studies reviewed was that whether they were cross-sectional or longitudinal in design, they were not powered to detect fracture prevalence or incidence, respectively. Evidence from a recent national prospective data collection in South Africa showed fracture rates largely mirror those observed elsewhere in the world, with White and mixed-race African populations having higher fracture rates than Black Africans [13••]. Whilst, within South Africa, fracture rates may be lowest in Black Africans, fracture outcomes are poorer, with much higher morbidity and mortality in Black South African women and men than is seen in other countries [15].

In Botswana, an albeit retrospective review of incident hospital data records showed much lower fracture rates in Botswanan women than has been reported in South African women, suggesting the epidemiology may differ across countries, although methodologies differ too [57]. Collection of robust fracture rates for hip and vertebral fracture, as well as associated risk factors, is much needed across the region. Notably, of 131 fracture liaison services surveyed globally in 2020, only one of those was in SSA; it was operational in South Africa [58].

## Conclusion

In conclusion, those in sub-Saharan Africa do not have equitable access to diagnostic and treatment options for osteoporosis to reduce future fragility fracture risk. Yet, osteoporosis and osteopenia are common amongst older Black African women, and to a lesser extent men. The demands osteoporosis and associated future fractures will place on already stretched healthcare systems must be given attention. Awareness is certainly increasing, with recognition of the importance of appropriate diagnostic and management pathways. It will be important to ensure that communities and stakeholders are fully consulted, as pathways are co-developed, to ensure practical context-specific solutions can be found. Overall, reprioritization towards care of ageing populations is a growing necessity, with equitable access to diagnostic services and provision of healthcare now a key goal for healthcare services, policymakers and governments.

## Supplementary Information

Below is the link to the electronic supplementary material.

Supplementary file1 (DOCX 36 KB)

## Data Availability

The data that support the findings of this study are available from the principal investigators of each cohort upon reasonable request.

## Footnotes

1 95–95-95 is the UNAIDS target for 2030, to achieve 95% of all people living with HIV to know their status; 95% of all people who know their HIV status to be receiving sustained treatment and 95% of all people having viral suppression.

2 For context. The Gambia and Ethiopia are classified as least-developed countries on the Development Assistance Committee list for overseas development aid. Tunisia and Zimbabwe are lower middle-income countries and South Africa and Botswana upper middle–income countries. For least developed countries, income is not declared, low-income per capita gross national income is < 1045USD; LMIC 1046–4095 USD; UMIC 2096–12,695 USD.

## Declarations

### Disclaimer

The views expressed are those of the authors and not necessarily those of Wellcome, the NIHR or the Department of Health and Social Care.
