# Supplementary material for: Disparities in the Prevalence of Osteoporosis and Osteopenia in Men and Women Living in Sub-Saharan Africa, the UK, and the USA
Source: Curr Osteoporos Rep. 2023 Jun 23;21(4):360–71. doi: 10.1007/s11914-023-00801-x (PMC10393839; doi:10.1007/s11914-023-00801-x)
Supplement: Supplementary file 1 — Supplementary file1 (DOCX 36 KB) [file 11914_2023_801_MOESM1_ESM.docx]

**COSR-D-23-00013R1**

**Disparities in the prevalence of osteoporosis and osteopenia in men and women living in Sub-Saharan Africa, the UK, and US**

**Kate A Ward PhD,** MRC Lifecourse Epidemiology Centre, University of Southampton, UK; MRC Unit The Gambia at London School of Hygiene and Tropical Medicine, Banjul, The Gambia, 0000-0001-7034-6750; kw@mrc.soton.ac.uk

*Appendices: Analytical approach, all analyses for this manuscript were conducted by Camillie Pearse and Tafadzwa Madinhire*

*T-score calculation*

T-scores were derived using NHANES III data. A T-score is a standard deviation score os it calculated per individual by subtracting the population mean and dividing by the standard deviation of the reference population:

T-score = (BMD femoral neck – 0.884) / 0.113

T-score = (BMD total hip – 0.971) / 0.114

*Country-specific burden was calculated by:*

The proportion of cases in the study cohort per 10-year age band was calculated and divided by the total number of participants and multiplying that by the number of people within each country. The total population figures were obtained from United Nations population estimates 2015 (UN World Population Prospects 2022 2022 [Available from: <https://population.un.org/wpp/>) (28)

*Population prevalence per 100,000 was calculated by:*

Calculating the proportion of cases in the study cohort per 10-year age band/ total number of participants and multiplying that by 100,000 to get the prevalence per 100,000.

Supplementary Table 1a: Femoral neck osteoporosis and osteopenia per 100,000 population

|  | Male |  | Female |  |
| --- | --- | --- | --- | --- |
| T-score | Osteopenia | Osteoporosis | Osteopenia | Osteoporosis |
| Cohort |  |  |  |  |
| **The Gambia (GamBAS) - 70+** | 31700 | 7700 | 62200 | 18500 |
| **The Gambia (GamBAS) - 60 - 69** | 24500 | 0 | 56700 | 2500 |
| **The Gambia (GamBAS) - 50 - 59** | 4400 | 0 | 33600 | 3200 |
| **The Gambia (GamBAS) 40 - 49** | 1100 | 0 | 6700 | 0 |
| **South Africa (Agincourt) - 70+** | 33300 | 0 | 57100 | 14300 |
| **South Africa (Agincourt) - 60 - 69** | 17100 | 7300 | 33300 | 2000 |
| **South Africa (Agincourt) - 50 - 59** | 13300 | 0 | 31000 | 2300 |
| **South Africa (Agincourt) - 40 - 49** | 8800 | 0 | 14600 | 0 |
| **South Africa (MASC) - 60 – 69** |  |  | 48500 | 23200 |
| **South Africa (MASC) - 50 - 59** |  |  | 44200 | 11400 |
| **South Africa (MASC) - 40 - 49** |  |  | 31300 | 5100 |
| **Zimbabwe (Menopause study) - 50 - 59** |  |  | 39000 | 3000 |
| **Zimbabwe (Menopause study) - 40 - 49** |  |  | 24000 | 2000 |
| **US (HealthABC -White) – 70+** | 46300 | 11400 | 51200 | 36200 |
| **US (HealthABC -White) – 60 - 69** | 31300 | 6300 | 57100 | 24100 |
| **US (HealthABC -Black) – 70+** | 31700 | 3100 | 46800 | 14800 |
| **US (HealthABC -Black) – 60-69** | 13640 | 0 | 48300 | 3500 |
| **UK (HCS) - 70+** | 19100 | 700 | 44800 | 5300 |
| **UK (HCS) 60 - 69** | 27200 | 900 | 52200 | 7300 |
| **UK (HCS) - 50 - 59** | 15400 | 0 | 0 | 0 |
|  |  |  |  |  |

Supplementary Table 1b: Total hip osteoporosis and osteopenia per 100,000 population

|  | Male |  | Female |  |
| --- | --- | --- | --- | --- |
| T-score | Osteopenia | Osteoporosis | Osteopenia | Osteoporosis |
| Cohort |  |  |  |  |
| **The Gambia (GamBAS) - 70+** | 31700 | 4800 | 40000 | 52500 |
| **The Gambia (GamBAS) - 60 - 69** | 33000 | 0 | 62500 | 22500 |
| **The Gambia (GamBAS) - 50 - 59** | 7000 | 0 | 40000 | 10400 |
| **The Gambia (GamBAS) 40 - 49** | 8800 | 0 | 12200 | 1100 |
| **South Africa (Agincourt) - 70+** | 21400 | 0 | 36700 | 16700 |
| **South Africa (Agincourt) - 60 - 69** | 9800 | 0 | 24600 | 4900 |
| **South Africa (Agincourt) - 50 - 59** | 5400 | 0 | 29400 | 2200 |
| **South Africa (Agincourt) - 40 - 49** | 4900 | 0 | 12000 | 0 |
| **South Africa (MASC) - 60 – 69** |  |  | 27300 | 4000 |
| **South Africa (MASC) - 50 - 59** |  |  | 17900 | 1600 |
| **South Africa (MASC) - 40 - 49** |  |  | 8700 | 1500 |
| **Zimbabwe (Menopause study) - 50 - 59** |  |  | 17000 | 0 |
| **Zimbabwe (Menopause study) - 40 - 49** |  |  | 6000 | 0 |
| **US (HealthABC -White) – 70+** | 27500 | 3900 | 50200 | 27200 |
| **US (HealthABC -White) – 60 - 69** | 18800 | 0 | 46400 | 21400 |
| **US (HealthABC -Black) – 70+** | 13800 | 1800 | 39000 | 14700 |
| **US (HealthABC -Black) – 60-69** | 9100 | 0 | 31000 | 3500 |
| **UK (HCS) - 70+** | 12700 | 1200 | 42700 | 7700 |
| **UK (HCS) 60 - 69** | 8000 | 300 | 35100 | 3800 |
| **UK (HCS) - 50 - 59** | 7700 | 0 | 0 | 0 |
|  |  |  |  |  |

Supplementary Table 2a: Country-specific burden of femoral neck osteoporosis and osteopenia

|  | Male |  |  | Female |  |  |
| --- | --- | --- | --- | --- | --- | --- |
| T-score | Normal | Osteopenia | Osteoporosis | Normal | Osteopenia | Osteoporosis |
| Cohort |  |  |  |  |  |  |
| **The Gambia (GamBAS) - 70+** | 10347 | 5412 | 1315 | 3849 | 12405 | 3690 |
| **The Gambia (GamBAS) - 60 - 69** | 18391 | 5968 | 0 | 10270 | 14272 | 629 |
| **The Gambia (GamBAS) - 50 - 59** | 47485 | 2186 | 0 | 33915 | 18031 | 1717 |
| **The Gambia (GamBAS) 40 - 49** | 82625 | 919 | 0 | 87119 | 6256 | 0 |
| **Zimbabwe (Menopause study) - 50 - 59** |  |  |  | 184002 | 123726 | 9517 |
| **Zimbabwe (Menopause study) - 40 - 49** |  |  |  | 402928 | 127240 | 10603 |
| **South Africa (Agincourt) - 70+** | 400610 | 200005 | 0 | 351531 | 701833 | 175766 |
| **South Africa (Agincourt) - 60 - 69** | 780346 | 176507 | 75351 | 1093746 | 562933 | 33810 |
| **South Africa (Agincourt) - 50 - 59** | 1538537 | 236015 | 0 | 1644746 | 764425 | 56715 |
| **South Africa (Agincourt) - 40 - 49** | 2761029 | 266415 | 0 | 2871370 | 490890 | 0 |
| **South Africa (MASC) - 60 – 69** |  |  |  | 478408 | 819887 | 392193 |
| **South Africa (MASC) - 50 - 59** |  |  |  | 1094853 | 1089921 | 281111 |
| **South Africa (MASC) - 40 - 49** |  |  |  | 2138398 | 1052388 | 171475 |
| **US (HealthABC -White) – 70+** | 3510585 | 1358608 | 3842555 | 5520691 | 946115 | 3903301 |
| **US (HealthABC -White) – 60 - 69** | 6346779 | 2098262 | 3183561 | 6372912 | 640781 | 2689793 |
| **US (HealthABC -Black) – 70+** | 1175485 | 899466 | 571516 | 1096224 | 55890 | 346669 |
| **US (HealthABC -Black) – 60-69** | 1908146 | 1168635 | 301379 | 1171060 | 0 | 84859 |
| **UK (HCS) - 70+** | 2807596 | 2248822 | 668642 | 2018982 | 24505 | 238853 |
| **UK (HCS) 60 - 69** | 2494071 | 1476829 | 943515 | 1903469 | 31219 | 266194 |
| **UK (HCS) - 50 - 59** | 3555741 |  | 647262 |  | 0 |  |
|  |  |  |  |  |  |  |

Supplementary Table 2b: Country-specific burden of total hip osteoporosis and osteopenia

|  | Male |  |  | Female |  |  |
| --- | --- | --- | --- | --- | --- | --- |
| T-score | Normal | Osteopenia | Osteoporosis | Normal | Osteopenia | Osteoporosis |
| Cohort |  |  |  |  |  |  |
| **The Gambia (GamBAS) - 70+** | 10842 | 5412 | 820 | 1496 | 7978 | 10471 |
| **The Gambia (GamBAS) - 60 - 69** | 16320 | 8038 | 0 | 3776 | 15732 | 5663 |
| **The Gambia (GamBAS) - 50 - 59** | 46194 | 3477 | 0 | 26617 | 21465 | 5581 |
| **The Gambia (GamBAS) 40 - 49** | 76192 | 7352 | 0 | 80957 | 11392 | 1027 |
| **Zimbabwe (Menopause study) - 50 - 59** |  |  |  | 263314 | 53932 | 0 |
| **Zimbabwe (Menopause study) - 40 - 49** |  |  |  | 498358 | 31810 | 0 |
| **South Africa (Agincourt) - 70+** | 472083 | 128532 | 0 | 572775 | 451091 | 205265 |
| **South Africa (Agincourt) - 60 - 69** | 931048 | 101156 | 0 | 1191795 | 415860 | 82834 |
| **South Africa (Agincourt) - 50 - 59** | 1678726 | 95826 | 0 | 1686666 | 724970 | 54249 |
| **South Africa (Agincourt) - 40 - 49** | 2879100 | 148345 | 0 | 2958789 | 403471 | 0 |
| **South Africa (MASC) - 60 – 69** |  |  |  | 1161366 | 461503 | 67620 |
| **South Africa (MASC) - 50 - 59** |  |  |  | 1985038 | 441394 | 39454 |
| **South Africa (MASC) - 40 - 49** |  |  |  | 3019310 | 292517 | 50434 |
| **US (HealthABC -White) – 70+** | 8258950 | 1912171 | 0 | 3593831 | 5178689 | 2388447 |
| **US (HealthABC -White) – 60 - 69** | 5693289 | 2282295 | 323671 | 2436867 | 5412865 | 2932867 |
| **US (HealthABC -Black) – 70+** | 2008459 | 201066.8 | 0 | 1084513 | 913520.4 | 344326.9 |
| **US (HealthABC -Black) – 60-69** | 1521639 | 248798.9 | 32452.03 | 1588083 | 751612 | 84859.42 |
| **UK (HCS) - 70+** | 3014140 | 444594.4 | 42008.92 | 2235302 | 1924342 | 347012.6 |
| **UK (HCS) 60 - 69** | 3180894 | 277504.4 | 10406.42 | 2228007 | 1279919 | 138566.7 |
| **UK (HCS) - 50 - 59** | 3879372 | 323631.2 | 0 |  |  |  |
|  |  |  |  |  |  |  |
|  |  |  |  |  |  |  |
